# Second Victim Experience: A Dynamic Process Conditioned by the Environment. A Qualitative Research

**DOI:** 10.3389/ijph.2024.1607399

**Published:** 2024-06-13

**Authors:** Maria Victoria Brunelli, Mariana Graciela Seisdedos, Maria Maluenda Martinez

**Affiliations:** ^1^ Escuela de Enfermería, Facultad de Ciencias Biomedicas, Universidad Austral, Buenos Aires, Argentina; ^2^ Departamento de Calidad y Seguridad del Paciente, Hospital Universitario Austral, Buenos Aires, Argentina

**Keywords:** psychological safety, second victim, healthcare workers, patient safety incidents, patient safety

## Abstract

**Objectives:**

When adverse events (AE) occur, there are different consequences for healthcare professionals. The environment in which professionals work can influence the experience. This study aims to explore the experiences of second victims (SV) among health professionals in Argentina.

**Methods:**

A phenomenological study was used with in-depth interviews with healthcare professionals. Audio recordings and verbatim transcriptions were analyzed independently for themes, subthemes, and codes.

**Results:**

Three main themes emerged from the analysis: navigating the experience, the environment, and the turning point. Subthemes were identified for navigating the experience to describe the process: receiving the impact, transition, and taking action.

**Conclusion:**

SVs undergo a process after an AE. The environment is part of this experience. It is a turning point in SVs’ professional and personal lives. Improving the psychological safety (PS) environment is essential for ensuring the safety of SVs.

## Introduction

Global healthcare is threatened by a shortage of healthcare workers [1]. In 2023, the WHO warned that 55 countries have urgent needs for healthcare professionals [2] and developed the Global Health Workforce Strategy 2030. It suggests, among other actions, improving job security [3], both physically and psychologically, for healthcare personnel.

Psychological safety (PS) is defined as a work environment where team members engage in dialogue, express opinions, and request help [4]. Work relationships are perceived as supportive and trustworthy [4], and teamwork is encouraged [5, 6].

Moreover, PS is closely related to patient safety [4–6]. First, it promotes error reporting [4, 7]. Derickson et al. reported that 20% more professionals were willing to report an error in psychologically safe hospitals than in other hospitals [7]. Second, PS is an ally in the care of professionals involved in a safety incident–known as second victims (SVs) [8]. Various authors affirm that SV care programs in psychologically safe environments contribute to their recovery [8, 9].

In 2022, the European Researchers’ Network Working on Second Victims (ERNST) Consortium presented a consensus on the definition of SVs, integrating existing evidence and clarifying previous concepts proposed by Wu and Scott [10]. Thus, a SV is defined as any healthcare worker who experiences a negative impact from direct or indirect involvement in an unexpected AE, unintentional medical error, or patient injury [10]. They often feel responsible for the patient’s outcome and perceived failure [10]. The prevalence varies between 13% and 78% according to different studies [11–13]. They experience emotional, physical, and/or professional disturbances and may desire to abandon the profession [8, 11–17]. For instance, Potura et al. demonstrated that 30.4% of Austrian pediatric reports exhibited self-doubt, 26.1% experienced flashbacks in similar professional settings, and 25.6% exhibited insomnia or an excessive need for sleep [12]. Furthermore, posttraumatic stress [18–21], suicide attempts [13, 22], and substance abuse [23, 24] have also been reported.

In addition, SVs greatly benefit from supportive measures [11, 25]. This support stems from various sources, including colleagues [11], supervisors [26, 27], and organizational channels [25, 28]. Research indicates that the extent of impact is negatively correlated with the perceived level of support, highlighting an inverse relationship [26]. Similarly, the safety culture within which professionals operate is significantly linked to the distress experienced by SVs [29].

For two decades, studies have shown the relevance of the SV phenomenon, mainly in Asia [15, 28, 30–32], the United States [14, 27, 33], and Europe [8, 11–13, 18, 34–37]. In 2009, Scott described the recovery trajectory of SVs through a qualitative study conducted with healthcare professionals in the United States [38]. Several factors influence the experience, such as the relationship between the patient and caregiver, past clinical experiences, and any perceived connection [38], all of which are influenced by work and sociocultural contexts.

In the Latin American context, there is less evidence of the SV phenomenon. It has been observed some quantitative studies in Argentina [26, 39], Brazil [40], Chile [25, 41], Colombia [42], and Mexico [43]. These studies suggest that the SV phenomenon is not uncommon in Latin American countries. However, no qualitative studies have been conducted that have allowed for a deeper understanding of the experiences of SVs in this socioeconomic, labor, and cultural context. Therefore, the objective of this study was to explore the experiences of SVs among healthcare professionals in Argentina.

## Methods

This phenomenological study was given access to the lived experience of healthcare professionals [44] who were involved in an AE to describe what the participants had in common regarding their SV experience [45]. The Consolidated Criteria for Reporting Qualitative Research (COREQ) guidelines [46] were used for this reporting.

### Population

The participants were healthcare professionals directly involved in an AE. The inclusion criteria was they work in highly complex hospital. Professionals who were on leave of absence were excluded. The sampling was purposive, selecting typical cases related to the phenomenon and a heterogeneous sample in terms of the participants’ professions. A minimum of 5 participants [45] were enrolled until theoretical data saturation was reached.

Phenomenological studies also take into account the context or situation in which the experience is lived to find the common denominator among the participants [45]. The professionals in this study worked in a private hospital that prioritizes patient safety in its mission. The hospital has international quality and safety accreditations in healthcare practices, voluntary incident reporting, and conducts morbidity-mortality meetings. While protocols are in place for the first victim, at the time of the study, there were no formal protocols described for SVs.

### Data Collection and Analysis

To access the participants, a professional who follows SVs as part of his job contacted the professionals and invited them to participate in the study. Those who agreed to participate were contacted by the principal investigator. Subsequently in-depth and semi structured face-to-face interviews were conducted using a developed question guide (Appendix). The questions were grouped into two main inquiries: “What was your experience as an SV?” and “What context or situation influenced your experience?” This approach aimed to elicit data that would lead to a description of the experience [45], thus facilitating an integrated understanding of the participants’ experiences.

The face-to-face interviews were audio recorded, and the data were transcribed verbatim, including field notes taken during the interviews.

Phenomenological studies typically involve two main movements: epoché and reduction [45]. Epoché is required to suspend personal beliefs [45]. Therefore, a written document was prepared beforehand to outline the researchers’ personal assumptions about the phenomenon being studied. The purpose of this step was to ensure the researchers were aware of their concepts and beliefs about the topic and to exclude them from the data analysis. In the second step, the researchers engaged in reflexivity with respect to the data [45] to capture the meaning of the experience. This process included delineating the quotations and units of meaning (codes) and determining the central theme for the units of meaning. Finally, in accordance with phenomenological requirements, all the themes were integrated into a particular structure [45].

ATLAS.ti version 22 software was used. Triangulation of the data analysis was performed by two researchers who independently analyzed the data. They met to discuss their initial codes and to identify commonalities in their analyses. Next, they reanalyzed the data individually and, in the subsequent joint session, agreed on the final emergent codes, organizing them into subthemes and themes. Finally, this analysis was presented to two other researchers for validation.

The study received approval from the Institutional Review Board (No. 19–017). All participants provided prior consent before participating in the interview sessions, which were conducted in person and lasted approximately 50 min each.

## Results

Seven participants were interviewed, including individuals from various healthcare professions, such as nurses, physicians, and surgical technologists, with diverse specialties, including internal medicine, surgery, cardiology, and anesthesia. The time since the AE varied, ranging from 6 months to 10 years.

During the interview analysis, it was noted that despite the dynamic and personal nature of the SV experience, all participants went through a process within a specific context or environment. Subthemes and codes were identified within each of the three themes (Table 1).

**TABLE 1:** Themes, subthemes and codes (Argentina, 2019).

Theme	Subtheme	Codes
Navigating the experience	Receiving the impact	Emotional response
		Physical response
		Professional insecurity
		Dispersion
		Paralysis
	Transition	Professional pause Rumination Dissociation
	Taking action	Learning Message protagonist Reference for others
Environment		Institutional culture Colleague support Supervisor support Institutional support Support outside of work
Turning point		

### Navigating the Experience

Navigating the experience encompasses the process that SVs go through. Three subthemes that make up this process delineate moments or stages: receiving the impact, transition, and taking action. Each subtheme includes various codes that define the states, actions, or characteristics of the participants. However, as noted above, the experience is dynamic and personal. Therefore, it is important to note that these moments are not rigid, and some codes are not limited to a single stage but may manifest across multiple stages or during the transition between them. Some quotes are shown in Table 2.

**TABLE 2:** Quotes of process (Argentina, 2019).

Subthemes	Codes	Quotes
Navigating the experience	Emotional response	• “*Upon exiting, my immediate impulse was to seek forgiveness from my team, repeatedly expressing remorse. I was overwhelmed with a profound sense of guilt*” (P5) • *“And every time, I do not know, if I had a problem for any reason, I do not know, if I misplaced a chair, it was… ‘no, because you made a mistake with a patient.’ And it had nothing to do with it. […] even though I had made a mistake, I did not want to be reminded of it all the time. Or else, they could have just removed me from my position and placed me somewhere else if they did not believe I was capable of continuing to work. […] And still, I felt like I was walking down the hallway and people were staring at me. Maybe it was just my imagination. That they were pointing at me.” (P5)*
	Physical response	• *“(…) Nightmare, you go to bed, you sleep well because sleep overcomes you but two hours later you wake up because you are having a nightmare. You are dreaming about the scalpel, the patient, the lawyer, the family; all this is (…)” (P2)*
	Professional insecurity	• *“All night* *[* *on guard], all night long. Then, I had to continue attending* *[* *to patients]. You know when your hands shake? You cannot think straight. I had no one to ask for help (…) My hands were shaking, I could not think, my whole body was shaking. I do not know who I asked for help, or if help even came. Honestly, I cannot tell you…"* (P7)
	Dispersion	• *“It’s like (…) you keep thinking and you’re there, absorbed in your thoughts, I do not know, you keep doing your tasks but with that in your mind.”* (P1)
	Paralysis	• *“Honestly, I was blocked, frozen. You know when they put you in the freezer and you have no reaction, not to black, not to white, not to good, not to anything. Frozen. I struggle; if I do not refer to the medical records, I have difficulty recalling things, as if I have blanks from the episode itself.” (P7)*
Transition	Professional pause	• *“And I told him that yes, that I also did not realize that day to say, ‘Look, today I’m not here, I do not know, have me do something else.’ Uh, I was not even aware of that, but I do remember it.”* (P6)
	Rumination	• *“Everything. (…) It was like watching a movie a hundred times in my head, playing repeatedly. ‘And what would I have done? And what would I have?’ And honestly, there’s not much I would have done differently.” (P7)*
	Dissociation	• *“That’s why I believe that […] you take everything else out of context, and I thought it could work, listen to me, it worked well, smoothly. Many times I thought, but it’s like being a theater actor, you know? Because you say, okay, you may have a very painful family drama, but you get on stage and act, or the experience acts for you.”* (P2)
Taking action	Learning	• *“After the event, what changed is that I do everything step by step (…) The pace at which we are pushed to work sometimes makes us do everything quickly and without thinking. So now, I put a brake on it. Even if my colleagues tell me to hurry up later, I do everything step by step. I do this, finish it, and then move on to the next thing. Because I notice a lot that happens, okay, hurry up, just write it down quickly and then move on. No, I do everything step by step, slowly. I’m not saying like a turtle, but I go at a pace, but I finish one thing and then do the next. That also helped me to slow down a bit, the rush we had while working.”* (P5)
	Message protagonist	• *“Well, what happened, if anyone wants to know, they can ask me. I was able to speak about it. I never denied it, I talked about it. I think the people who found out from me did not make assumptions or guesses. To those who came to ask me, I explained: ‘I do not feel good about it, it’s not pleasant, it’s not good for everyone to talk about it.’ And in fact, what they recommended to me, I told them: ‘Look, they do not want me to talk about this, they want it to stay quiet because then it turns into a big pot of gossip, and that’s not good either’ (…) At least I talked about it; my colleagues who came to ask me, I told them what the situation was.” (P1)*
	Reference for others	• *“To help him, I would meet with him, ensure he has specialized counseling to see how he’s managing this trauma because it is trauma (…) talking with the person, asking them what they want, dispelling clouds, uh-huh. And then (…) I would take him to a psychological interview where he could express many things in a comfortable environment and decide what he wants, and then the return has to be supervised, accompanied, that transition between being alone and being accompanied.” (P2)*

#### Receiving the Impact

The process begins when SVs express various emotions, thoughts, and experiences that indicate that they are receiving the impact. These expressions are often involuntary and intrusive.

The emotional response is a code of this initial stage. It involves the emotional state, which manifests as an emotional storm and is expressed as a manifestation of a number of simultaneous or nonsimultaneous emotions, without any one of them dominating, or when people find it difficult to name their emotions. For example, one participant expressed what he felt upon returning to work after the AE, stating, “*It’s like being robbed at home and then going back in knowing the thief is not there, but it’s a feeling…*" (P2). Terms such as “*devastating, terrible, unforgettable, could not do anything, was stunned, or could not believe it*” (P3) reflect the emotional state of the participants. In other cases, SVs are able to directly identify the emotion, using terms such as guilt, stigmatization and loneliness. Guilt emerges as a pervasive emotion among SVs. They struggle with feelings of culpability for the effects of the AE on the patient. In addition, SVs experience a sense of responsibility for their team, perceiving their inability to prevent the AE as a source of harm not only to the patient but also to the teamwork of their team. Additionally, perception of stigmatization the SVs perceive themselves as being scrutinized and judged, characterized by the label “*you made a medical error*” (P5). This phenomenon is particularly prevalent in the professional sphere but also extends to their family or social circles, where some professionals opt for silence out of fear of possible labeling or public exposure of their situation. Finally, feelings of loneliness are often present in SVs in response to a perceived lack of understanding of their environment.

Moreover, there are physical manifestations or consequences of receiving the impact. Participants mentioned cases of insomnia, nightmares, eating disorders, and even disease diagnoses, all of which are considered aftereffects of the traumatic event.

Furthermore, participants expressed a pervasive sense of professional insecurity. They harbor doubts about their ability to provide patient care, fear the possibility of repeating the error, and perceive themselves as inadequately equipped for their professional duties. These feelings persist not only in the immediate aftermath of the incident, when they are required to resume patient care, but also extends into the subsequent days and weeks following their return to the workplace.

Another code identified in this stage is dispersion. This code indicates that SVs demonstrated an inability to concentrate on tasks, making them more susceptible to new events.

Finally, another situation they experience when receiving the impact is paralysis, which hinders or prevents them from continuing with their tasks.

#### Transition

The transition state is characterized by the reactions that SVs exhibit as a means of coping with the situation. These include professional pauses, rumination, and dissociation.

A professional pause represents the desire to distance oneself from the environment in which the AE occurred.

In addition, SVs expressed that they experienced constant rumination in an intrusive and involuntary manner. This incessant rumination allowed them to replay, step-by-step, the actions they had taken before the AE. However, it also interfered with their ability to concentrate on other activities.

Finally, SVs may experience dissociation. Here, an analogy with a theater is drawn, comparing SVs with actors playing roles that are not real. In this way, the need to continue working does not allow them to acknowledge or express their feelings. Consequently, to continue their duties, they must assume a fictional persona because “*the show must go on*” (P7). At the same time, dissociation allows SVs to jump into action to persevere. This illustrates that some of the codes cannot be confined to a single moment in the process and that this is a dynamic process.

#### Taking Action

Taking action represents the final stage of the process. It encompasses the internal and/or external actions experienced by SVs that indicate movement toward exiting the situation.

This stage is characterized by actions aimed at remedying the situation, learning from the event, and contributing insights gained from their experience. The codes that comprise this stage include learning, being a messenger, and striving to be a role model for others.

Learning occurs at two levels. First, there are lessons related to the incident itself (within the work environment). Second, there are lessons related to the lives of the participants that can be translated into attitudes or values they will adopt in the future. For example, empathy, humanity, understanding, and support for colleagues are among the lessons that SVs share in their narratives.

In addition, SVs express a desire to be protagonists of the message. In doing so, they hoped to be the ones to communicate the event to those around them. They wanted to avoid the stigmatization they felt they were subjected to and the misinformation surrounding the event.

Finally, the participants want to serve as a reference for others, especially for those who may have a similar experience. SVs feel that the incident provides them with valuable experience.

### Environment

The experience of SVs is significantly influenced by their surrounding environment, including both their workplace and personal domains. Various factors within the professional setting shape their experience, such as the institutional culture, support from colleagues and supervisors, and institutional backing. Meanwhile, in their personal lives, SVs may rely on support from family and friends. In both domains, the SV actively seek or passively receive support from individuals in their social circle. Table 3 shows some quotes related to the environment codes.

**TABLE 3:** Quotes of environment (Argentina, 2019).

Codes	Quotes
Institutional culture	• *“* *[* *The patient was told that there had been a mistake] (…) the same day and the family was also told the same day (…) they told her what the possibilities were of what she could have. And (…) … the first thing the patient said the next day was that thanks for having told her the truth, that she was not lied to (…)* (P5) • *“What were the things that worked for me? The first is to reconstruct the event. Talking to all the members “* *[* *of the team] about why it happened. Secondly, not to look for people to blame. It is neither the place nor the moment nor the mood to do so (…) It is the moment to say what do we do? Which is different from… I mean, if I were on the Titanic I would not be worried about why the water got into… but, how do we save ourselves? (…) Third, (…) I would try to tell the truth (…) to the family. Because the family takes well a guy who tells the truth. (…)* (P4)
Colleague support	• *“Yes, and some colleagues also wanted to lend me a hand, reaffirming their trust in me. They would call me to work outside when I no longer wanted to work externally. Interviewer: Well, did that help you? Interviewee: Yes, the affection, the attitude.”* (P2)
Supervisor support	• *“Yes, mm (…) we talked to the two afternoon coordinators (…) I felt very accused because one said ‘I cannot believe that you’re here all the time.’ I do not know (…) ‘That every mistake you come and tell us, I cannot believe you did not tell us anything (…).' I told her (…) it was not intentional to hide the information and cover it up (…) The feeling was one of accusation; that our intention was to cover it up, and it did not make sense to me to cover something like that. Um, I do not know (…) I remember both of us were crying (…); I felt that I did not have the support (…) it was like we were in the hot seat (…). It was a freezing office. Um (…) they made us go wash our faces and take some time and (…) then go back to work (silence). Horrible; horrible.”* (P6)
Support outside of work	• *“Perhaps, I felt that my friend, sharing the same profession, could empathize with me at that moment and understand everything. And I think I was seeking fundamental emotional support. A comforting gesture, a soothing balm, a caress, a kiss (…) And with my husband, you see, he was first an instrument technician and now he’s a dentist. And he tells me, ‘Don’t worry because I was a wreck during my first week.’ (…) It was a torment, morning, noon, and night. I did not eat, I did not sleep, nothing. Until he says to me (…) you have to turn the page and be convinced. I know how you work, you did everything you had to do. Without being there inside, I know you did. Enough, he says to me. Enough. I think he gave me a wake-up call. Enough because we cannot go on like this as a family.”* (P7)

The behaviors and responses of SV provide insight into the institutional culture in which they are embedded. In this regard, participants expressed an interest in prioritizing transparency in communication, not only among team members but also with patients and their families. Moreover, they sought to investigate the root causes of the AE, and in their speeches, the reporting of the event appears recurrently as a routine task they perform. In addition, the learning from the of experience that SVs expressed its a characteristics of a robust safety culture.

Colleague support played a crucial role in the SV experience. Some professionals viewed this support positively, especially when it reinforced their confidence in their professional abilities or when the team collectively assumed responsibility for the AE.

The SVs had varying perceptions of their supervisors’ responses. In some cases, support from supervisors was perceived as lacking in empathy and understanding toward them as individuals. The participants did not indicate whether supervisors sought to analyze how the AE occurred but rather emphasized how they felt they were treated. Some viewed demands to continue working or imperative instructions to avoid repeating a situation as negative support. However, others valued supervisors who show concern for their wellbeing.

In a secondary role, institutional support comes into play, expressed at times through information provided about the AE and the institutional management offered to SVs (e.g., legal support and professional support). Institutional support was seen as the backing of various individuals within the institution (fulfilling management, commercial, or legal roles) who, although not previously known to them, are felt by the SV as having supported them.

Finally, but equally significant, is the support outside of work sought by the SV. Some individuals look for support talking with their family or friends.

### Turning Point

Ultimately, the study results showed that the AE marked a before and after moment in the professional lives of the participants; it was a turning point. On the one hand, terms such as *‘institutional proscription’* (P2), *‘it’s the end of my career’* (P2), and *‘it was the worst thing that happened to me in my career’* (P7) indicate that the SVs feel it is the end of their professional development. On the other hand, they use metaphors for natural disasters (such as *earthquake, tsunami, upheaval*). (Table 4).

**TABLE 4:** Turing Point quotes (Argentina, 2019).

*Turning Point*	• *"(…) At one point* *[* *a colleague] was as dismayed as I was, and I remember a dialogue the next day, over the phone, where he spontaneously said to me, ‘It’s like the end of your career,’ and he started to cry. Yes, generally when I mention my institutional proscription, it reflects that. It’s very strong. But yes, this makes you rethink whether you are useful or not. For me, it was a before and after.” (P2)* • *“I was devastated (…) a train ran over me. It was in my career (…) the worst thing I had, but by far. The worst thing I had. As far as, you know, when you feel like everything you have done is not enough? And then something happened that had never happened to me before.“* (P7)

## Discussion

The professionals involved in an AE experience a pivotal moment in their personal and professional lives. They are going through a dynamic process influenced by their environment. PS is fundamental to the recovery of SVs.

The process consists of different stages that professionals go through. This journey is clear and distinct, yet at the same time unique and personal, as each participant may experience diverse ways of living through each stage in terms of intensity and the order in which different states occur. In a different cultural, social, and work context, Scott et al. identified stages in the recovery of SVs [38]. Although the stages found in this study are not identical to those presented by Scott, common characteristics can be identified. The environment plays a fundamental role in the recovery of SVs. When colleagues or supervisors do not provide support to professionals, coping becomes more difficult. As a result, communication and teamwork break down and PS is weakened [8].

As they experience the impact, they manifest an emotional storm that reflects not only an awareness of the gravity of the event but also a strong vocational sense. Precisely because the experience of the phenomenon and its impact are so profound, they cannot identify a single emotion but rather a mixture of them, and these emotions are sometimes contradictory. Similarly, Scott proposes emotional chaos [38] as the first stage, wherein professionals attempt to reflect on what occurred. Additionally, consistent with other studies [13, 17, 31], professionals exhibited profound guilt for the patient, expressing the professional commitment and responsibility they hold as a healthcare professional.

Negative feelings such as guilt and fear lead to the stigmatization experienced by professionals. It is important to consider that this stigma originates from the work environment (colleagues, supervisors, etc.) but also likely from the SVs themselves. That is, they feel the weight of their mistake, and it is highly possible that they will continue to reproach themselves for it, regardless of the reaction from their environment. In these cases, the attitudes of colleagues become even more important in mitigating stigmatization and fostering a psychologically safe environment for SVs [8, 47].

The loneliness experienced by professionals is closely linked to a lack of understanding or support in the work environment. Consistently, various studies have revealed a lack of empathy and understanding toward SVs [13, 19, 47]. Bass et al., in a quantitative study, argued that the majority of professionals reported not having received training in emotional strategies to support colleagues. However, these same participants had sought support from their colleagues when they were involved in an AE [19]. In this context, the experience of loneliness may be intensified by the expectation of increased empathy and support from individuals who have experienced similar adversity. Therefore, it is necessary to train professionals to provide emotional first aid. Undoubtedly, the implementation of these training programs contributes to psychologically safe work environments [4, 47, 48].

Professional pauses and rumination mark a transition stage in which professionals seem to need space for reflection to understand and analyze what happened and reflecting a significant impact of the AE on the SV. In these transitions appears that after the initial shock wears off, as SVs consciously or unconsciously seek strategies to navigate the event. In these sense, in many situations, work demands and brief times of attention to respond to economic imperatives, among others, clash with this need of SVs and consequently with patient safety. Conversely, when organizations promote PS interventions, such as debriefing spaces [11, 48], they have a significant impact on SVs [16].

Additionally, the learning process and the desire to offer recommendations (in clinical practice to prevent future AEs or for colleagues going through the same situation) indicate that SVs are taking action. Now, they can serve as a reference by making recommendations on how to handle SVs. This finding is similar to that of Scott et al. who, in their final stage [38], found that SVs had three possible final outcomes. One of them was able to give new meaning to the experience [38]. Also learning is an intervention that contributes to creating a psychologically safe environment [48] and facilitates the recovery of SVs [38].

Research into the environment is a constant concern in the recovery process of healthcare professionals encompassing both the workplace (institutional culture and support from colleagues, supervisors, and the institution) and extralaboral factors.

From the environment, the SVs expect to receive support from colleagues and supervisors. Peer support fosters teamwork, which is critical for patient safety and psychological safety [5]. They also expect their immediate superiors to demonstrate humanity, understanding, and support. If healthcare professionals are not supported by their colleagues and supervisors, they rarely perceive that they are in a psychologically safe environment. Consequently, they lack confidence, avoid dialogue, and refrain from expressing their opinions. This breakdown in communication and teamwork leads to a loss of PS [5, 6, 8].

Finally, the inclination to seek assistance from family or social circles underscores the SV’s aspiration to avoid judgment and find solace in environments characterized by stronger interpersonal bonds. This tendency may stem from the profound uncertainty engendered by the experience, prompting professionals to seek refuge in environments reminiscent of prevent normalcy. Additionally, this underscores the paramount importance of emotional support, as evidenced by the spontaneous expression of this theme by the majority of the participants, without direct questions about this aspect.

This study has several limitations. First, generalizing the findings may be limited due to the specific context in which the study was conducted. Additionally, while efforts were made to conduct comprehensive interviews, participants may have withheld or forgotten some of their experiences.

Despite these limitations, this study provides a comprehensive understanding of the experiences of SVs in a Latin American culture. These findings enables the implementation of actions aimed at strengthening the PS of the workplace. Specifically, educational interventions focused on improving teamwork through simulation and debriefing; promoting interproffessional communication, trust, and support for SVs; and designing institutional-level SV response programs involving colleagues, supervisors, patient safety committees, and legal entities are important actions for strengthening workplace PS. Furthermore, it highlights the need for further research to determine how psychologically safe environments contribute to SVs recovery.

In conclusion, SVs undergo a process following an AE that is influenced by the workplace environment. This experience represents a turning point not only in their professional lives but also in their personal lives.

## Appendix: Second Victim Question Guide

### What was your experience as an SV?

What was your participation in the event/How did you feel about your participation?

What was the outcome of the event for the patient?

Do you remember how you felt immediately after the event?

Did you have any needs (emotional, physical, professional)?

Did you express or tell anyone about these needs, and did you receive a response?

If you had a response: What response did you get, from whom, and did the response meet your need?

Did you express a need that was not met at the time? What happened to it?

Do you think the incident had an impact on your professional life? Did anything change in you? What? How?

If you perceive a change: Do you think this change is good, bad, or neutral?

On a professional level, when you returned to see patients after the incident, did you feel different (sometimes people feel fear, insecurity, embarrassment, inability to continue seeing patients, desire not to return to work)? Did this happen to you? Have you experienced any other feelings related to your professional performance that we have not discussed?

Can you identify three feelings or emotions you experienced at the time? How long did they last?

### What context or situation influenced your experience?

How did you perceive your colleagues’ reactions? How did this reaction affect you (did it create positive or negative feelings?) Were you able to express it to them?

If you discussed the incident with a supervisor/coordinator or boss: How did you perceive your supervisor’s reaction to you? How did this reaction affect you (did it create positive or negative feelings?) Were you able to express it to them?

If you could go back in time, how would you have liked to have been treated by your colleagues? And by your supervisor/boss/coordinator?

Do you think the work environment would be different for you today? If so, how? Why do you think this change has occurred? How do you perceive it?

Do you highlight any behavior that was useful to you at the time?

If you are worried or stressed about a work-related problem, how do you usually handle this type of situation?

Whom do you usually turn to for advice or support in work-related situations?

## References

[1] The Lancet Global H. Health-Care Workers Must Be Trained and Retained. Lancet Glob Health (2023) 11(5):e629. 10.1016/s2214-109x(23)00172-9 37061296

[2] World Health Organization. WHO Health Workforce Support and Safeguards List (2023). Available from: https://www.who.int/publications/i/item/9789240069787 (Accessed March 24, 2024). 10.2471/BLT.23.290191 PMC10225944 37265683

[3] World Health Organization. Global Strategy on Human Resources for Health: Workforce 2030 (2016). Available from: https://www.who.int/publications/i/item/9789241511131 (Accessed March 24, 2024).

[4] Kumar S. Psychological Safety: What It Is, Why Teams Need It, and How to Make It Flourish. Chest (2023) 165(4):942–9. 10.1016/j.chest.2023.11.016 37977265

[5] Arad D Finkelstein A Rozenblum R Magnezi R. Patient Safety and Staff Psychological Safety: A Mixed Methods Study on Aspects of Teamwork in the Operating Room. Front Public Health (2022) 10:1060473. 10.3389/fpubh.2022.1060473 36620282 PMC9816421

[6] Han JH Roh YS. Teamwork, Psychological Safety, and Patient Safety Competency Among Emergency Nurses. Int Emerg Nurs (2020) 51:100892. 10.1016/j.ienj.2020.100892 32659674

[7] Derickson R Fishman J Osatuke K Teclaw R Ramsel D. Psychological Safety and Error Reporting Within Veterans Health Administration Hospitals. J Patient Saf (2024) 11(1):60–6. 10.1097/PTS.0000000000000082 24583957

[8] Nijs K Seys D Coppens S Van De Velde M Vanhaecht K. Second Victim Support Structures in Anaesthesia: A Cross-Sectional Survey in Belgian Anaesthesiologists. Int J Qual Health Care (2021) 33(2):mzab058. 10.1093/intqhc/mzab058 33760071

[9] Merandi J Liao N Lewe D Morvay S Stewart B Catt C Deployment of a Second Victim Peer Support Program: A Replication Study. Pediatr Qual Saf (2017) 2(4):e031. 10.1097/pq9.0000000000000031 30229168 PMC6132481

[10] Vanhaecht K Seys D Russotto S Strametz R Mira J Sigurgeirsdóttir S An Evidence and Consensus-Based Definition of Second Victim: A Strategic Topic in Healthcare Quality, Patient Safety, Person-Centeredness and Human Resource Management. Int J Environ Res Public Health (2022) 19(24):16869. 10.3390/ijerph192416869 36554750 PMC9779047

[11] Marung H Strametz R Roesner H Reifferscheid F Petzina R Klemm V Second Victims Among German Emergency Medical Services Physicians (SeViD-III-Study). Int J Environ Res Public Health (2023) 20(5):4267. 10.3390/ijerph20054267 36901278 PMC10001835

[12] Potura E Klemm V Roesner H Sitter B Huscsava H Trifunovic-Koenig M Second Victims Among Austrian Pediatricians (SeViD-A1 Study). Healthcare (2023) 11(18):2501. 10.3390/healthcare11182501 37761698 PMC10531173

[13] Magaldi M Perdomo JM Lopez-Baamonde M Chanza M Sanchez D Gomar C. Second Victim Phenomenon in a Surgical Area: Online Survey. Rev Esp Anestesiol Reanim Engl Ed (2021) S0034-9356:30320–0. 10.1016/j.redar.2020.11.009 34764069

[14] Burlison J Scott S Browne E Thompson S Hoffman J. The Second Victim Experience and Support Tool: Validation of an Organizational Resource for Assessing Second Victim Effects and the Quality of Support Resources. J Patient Saf (2017) 13(2):93–102. 10.1097/PTS.0000000000000129 25162208 PMC4342309

[15] Choi EY Pyo J Lee W Jang SG Park YK Ock M Nurses’ Experiences of Patient Safety Incidents in Korea: A Cross-Sectional Study. BMJ Open (2020) 10(10):e037741. 10.1136/bmjopen-2020-037741 PMC7783619 33130562

[16] Klemm V Rösner H Bushuven S Strametz R. The Second Victim Phenomenon-What Personnel in Anesthesiology Should Know About It. Anaesthesiologie (2023) 72(11):803–8. 10.1007/s00101-023-01337-6 37688607

[17] Busch IM Moretti F Purgato M Barbui C Wu AW Rimondini M. Psychological and Psychosomatic Symptoms of Second Victims of Adverse Events: A Systematic Review and Meta-Analysis. J Patient Saf (2020) 16(2):e61–74. 10.1097/PTS.0000000000000589 30921046 PMC7386870

[18] Wahlberg Å Andreen Sachs M Johannesson K Hallberg G Jonsson M Skoog Svanberg A Post-Traumatic Stress Symptoms in Swedish Obstetricians and Midwives After Severe Obstetric Events: A Cross-Sectional Retrospective Survey. BJOG: Int J Obstet Gynaecol (2017) 124(8):1264–71. 10.1111/1471-0528.14259 27562912

[19] Baas MAM Scheepstra KWF Stramrood CAI Evers R Dijksman LM Van Pampus MG. Work-Related Adverse Events Leaving Their Mark: A Cross-Sectional Study Among Dutch Gynecologists. BMC Psychiatry (2018) 18(1):73. 10.1186/s12888-018-1659-1 29566667 PMC5863895

[20] Kerkman T Dijksman LM Baas MAM Evers R van Pampus MG Stramrood CAI. Traumatic Experiences and the Midwifery Profession: A Cross-Sectional Study Among Dutch Midwives. J Midwifery Womens Health (2019) 64(4):435–42. 10.1111/jmwh.12946 30888739 PMC6767047

[21] Kruper A Domeyer-Klenske A Treat R Pilarski A Kaljo K. Secondary Traumatic Stress in Ob-Gyn: A Mixed Methods Analysis Assessing Physician Impact and Needs. J Surg Educ (2021) 78(3):1024–34. 10.1016/j.jsurg.2020.08.038 32948508

[22] Grissinger M. Too Many Abandon the “Second Victims” of Medical Errors. P T (2014) 39(9):591–2. 25210409 PMC4159062

[23] Van Gerven E Vander Elst T Vandenbroeck S Dierickx S Euwema M Sermeus W Increased Risk of Burnout for Physicians and Nurses Involved in a Patient Safety Incident. Med Care (2016) 54(10):937–43. 10.1097/mlr.0000000000000582 27213542

[24] Stehman CR Testo Z Gershaw RS Kellogg AR. Burnout, Drop Out, Suicide: Physician Loss in Emergency Medicine, Part I. West J Emerg Med (2019) 20(3):485–94. 10.5811/westjem.2019.4.40970 31123550 PMC6526882

[25] Kappes M Delgado-Hito P Contreras VR Romero-García M. Prevalence of the Second Victim Phenomenon Among Intensive Care Unit Nurses and the Support provided by Their Organizations. Nurs Crit Care (2023) 28(6):1022–30. 10.1111/nicc.12967 37614030

[26] Brunelli MV Estrada S Celano C Bandriwskyj C Riquelme RJ Ortega A Second Victim Experience and Support From Health Professionals. Medicina (2023) 83(6):918–26. 38117711

[27] Burlison JD Quillivan RR Scott SD Johnson S Hoffman JM. The Effects of the Second Victim Phenomenon on Work-Related Outcomes: Connecting Self-Reported Caregiver Distress to Turnover Intentions and Absenteeism. J Patient Saf (2021) 17(3):195–9. 10.1097/pts.0000000000000301 27811593 PMC5413437

[28] Mok WQ Chin GF Yap SF Wang W. A Cross-Sectional Survey on Nurses' Second Victim Experience and Quality of Support Resources in Singapore. J Nurs Manag (2020) 28(2):286–93. 10.1111/jonm.12920 31789437

[29] Quillivan RR Burlison JD Browne EK Scott SD Hoffman JM. Patient Safety Culture and the Second Victim Phenomenon: Connecting Culture to Staff Distress in Nurses. Jt Comm J Qual Patient Saf (2016) 42(8):377–86. 10.1016/s1553-7250(16)42053-2 27456420 PMC5333492

[30] Luk LA Lee FKI Lam CS Soh Y Wong YYM Lui WSW. Healthcare Professional Experiences of Clinical Incident in Hong Kong: A Qualitative Study. Risk Manag Healthc Pol (2021) 14:947–57. 10.2147/rmhp.s292875 PMC7953886 33727871

[31] Cho DB Lee W Cha JM Kim JH Kim J Kang SB Second Victim Experience and Perception Discordance of the Colonoscopic Perforation. Dig Dis Sci (2022) 67(7):2857–65. 10.1007/s10620-021-07107-x 34283361

[32] Pyo J Choi EY Lee W Jang SG Park YK Ock M Physicians' Difficulties Due to Patient Safety Incidents in Korea: A Cross-Sectional Study. J Korean Med Sci (2020) 35(17):e118. 10.3346/jkms.2020.35.e118 32356419 PMC7200176

[33] Finney RE Torbenson VE Riggan KA Weaver AL Long ME Allyse MA Second Victim Experiences of Nurses in Obstetrics and Gynaecology: A Second Victim Experience and Support Tool Survey. J Nurs Manage (2021) 29(4):642–52. 10.1111/jonm.13198 PMC8079544 33113207

[34] Gómez-Durán EL Arimany-Manso J. Healthcare Professional as Second Victim in Healthcare Injuries. Med Clin (Barc) (2020) 154(3):98–100. 10.1016/j.medcli.2019.09.005 31780216

[35] Carrillo I Tella S Strametz R Vanhaecht K Panella M Guerra-Paiva S Studies on the Second Victim Phenomenon and Other Related Topics in the Pan-European Environment: The Experience of ERNST Consortium Members. J Patient Saf Risk Manage (2022) 27(2):59–65. 10.1177/25160435221076985

[36] Vanhaecht K Seys D Schouten L Bruyneel L Coeckelberghs E Panella M Duration of Second Victim Symptoms in the Aftermath of a Patient Safety Incident and Association With the Level of Patient Harm: A Cross-Sectional Study in the Netherlands. BMJ Open (2019) 9(7):e029923. 10.1136/bmjopen-2019-029923 PMC6624045 31292185

[37] Van Gerven E Deweer D Scott SD Panella M Euwema M Sermeus W Personal, Situational and Organizational Aspects That Influence the Impact of Patient Safety Incidents: A Qualitative Study. Rev Calid Asist (2016) 31(Suppl. 2):34–46. 10.1016/j.cali.2016.02.003 27106771

[38] Scott SD Hirschinger LE Cox KR McCoig M Brandt J Hall LW. The Natural History of Recovery for the Healthcare Provider "Second Victim" After Adverse Patient Events. Qual Saf Health Care (2009) 18(5):325–30. 10.1136/qshc.2009.032870 19812092

[39] Brunelli MV Estrada S Celano C. Cross-Cultural Adaptation and Psychometric Evaluation of a Second Victim Experience and Support Tool (SVEST). J Patient Saf (2021) 17(8):e1401–e1405. 10.1097/pts.0000000000000497 29733300

[40] Sordi LPD Lourenção DCDA Gallasch CH Baptista PCP. The Second Victim Experience: Cross-Cultural Adaptation of an Instrument for the Brazilian Context. Revista Gaúcha de Enfermagem (2022) 43:e20210010. 10.1590/1983-1447.2022.20210010.en 35920520

[41] Mallea Salazar F Ibaceta Reinoso I Vejar Reyes C. Second Victims: Perceived Support Quality and Its Relationship With the Consequences of the Adverse Event. J Healthc Qual Res (2022) 37(2):117–24. 10.1016/j.jhqr.2021.09.002 34736894

[42] Flórez F López L Bernal C. Prevalence of Adverse Events and Their Manifestations in Health Professionals as Second Victims. Biomedica (2022) 42(1):184–95. 10.7705/biomedica.6169 35471180 PMC9084615

[43] Jiménez FEY Alayola SA Mancebo HA Campos CM. Eventos adversos y burnout en profesionales de una clínica de atención primaria. Rev CONAMED (2018) 23(2):66–72.

[44] Van Manen M. Writing in the Dark: Phenomenological Studies in Interpretive Inquiry. New York: Routledge (2016).

[45] Creswell JW. Qualitative Inquiry and Research Design. 3th ed. London: Sage (2013).

[46] Tong A Sainsbury P Craig J. Consolidated Criteria for Reporting Qualitative Research (COREQ): A 32-Item Checklist for Interviews and Focus Groups. Int J Qual Health Care (2007) 19(6):349–57. 10.1093/intqhc/mzm042 17872937

[47] O'Meara S D'Arcy F Dowling C Walsh K. The Psychological Impact of Adverse Events on Urology Trainees. Ir J Med Sci (2023) 192(4):1819–24. 10.1007/s11845-022-03202-8 36329289 PMC9633123

[48] O'Donovan R McAuliffe E. A Systematic Review Exploring the Content and Outcomes of Interventions to Improve Psychological Safety, Speaking up and Voice Behaviour. BMC Health Serv Res (2020) 20(1):101. 10.1186/s12913-020-4931-2 32041595 PMC7011517

